# Gender differences in attentional processes and attractiveness evaluation models during gait observation

**DOI:** 10.3389/fpsyg.2025.1451331

**Published:** 2025-03-26

**Authors:** Hiroko Tanabe, Kota Yamamoto

**Affiliations:** ^1^Graduate School of Humanities and Human Sciences, Hokkaido University, Sapporo, Japan; ^2^School of Humanities, Hokusei Gakuen University, Sapporo, Japan

**Keywords:** gender differences, gait, physical attractiveness, gaze behavior, gait animation, attractiveness perception

## Abstract

**Introduction:**

Physical attractiveness plays a crucial role in building interpersonal relationships and in daily communication. Attractiveness is perceived through nonverbal information regarding one’s morphological features, posture, movement, and behavior. Selective pressures throughout our species’ evolutionary history have shaped sex differences in the evaluation of physical attractiveness. However, research on the process of body attractiveness perception has been limited to static information involving body images. Therefore, a better understanding of the attractiveness perception process in the real world requires an appreciation of the attractiveness perception mechanism of physical movement.

**Methods:**

This study examined the attractiveness perception of 30-s walking animations, as well as gender differences in gaze behavior and statistical models of attractiveness evaluation. We recruited 16 men and 17 women and made gender comparisons of fixation ratio to each gaze area (head, trunk, hip, leg, and others). Furthermore, the standardized estimates of the statistical models were qualitatively compared between male and female observers.

**Results:**

Male observers were highly fixated on the walkers’ trunk, whereas female observers tended to shift their attention from the trunk to the legs, especially when observing high-preference animations. The statistical model for attractiveness evaluation, which used gait parameters for each gender, showed the tendency that when assessing attractiveness, male observers placed greater weight on the walkers’ trunk silhouette, whereas female observers prioritized parameters requiring whole-body observation.

**Discussion:**

Gender differences in gaze behavior were observed in the assessment and perception of human movement attractiveness; such differences may reflect the evaluation model for each gender. The results suggest that men assess female gait attractiveness based on observations of the reproductive regions of the female body. In contrast, women perceive other women as potential competitors and assess female gait attractiveness based on beauty standards, which are shaped by sociocultural environments and the walker’s psychological state. Our findings are the first step toward understanding the process of perceiving the attractiveness of physical movement and are expected to help generate attractive biological motions.

## 1 Introduction

In daily interpersonal communication, physical appearance and nonverbal behavior are important information for one’s perception of others. People value physical attractiveness over personality at the early stages of their interpersonal relationships (Heilman and Stopeck, 1985; Sabatelli and Rubin, 1986; Bixler and Nix-Rice, 1997), nonverbally increasing the immediacy between two individuals. Physical attractiveness works in two ways between physical characteristics (i.e., what is attractive) and the observers’ cognitive processes (i.e., how they feel attractiveness). Additionally, when an observer perceives attractiveness, their preferences for bodily appearance are closely linked to their attention. For example, Shimojo et al. (2003) discovered the gaze cascade effect, in which an observer’s attention shifts to an attractive face when they view two facial images simultaneously, suggesting that their attention is guided by their preferences, which in turn reinforces their preferences. Faces with high attractiveness are gazed at for longer periods (Leder et al., 2016). In such an attractiveness perception process, morphological features associated with physical attractiveness are identified and capture visual attention within 1 s of observation (Nummenmaa et al., 2012). For instance, when a woman observes body images of men with different morphological types, less attractive endomorphs focused on the lower back, where the adipose tissue is clearly visible, whereas more attractive mesomorphs focused on the entire back, where muscle tissue is distributed (Dixson et al., 2014). However, several studies on body attractiveness and its perception have been limited to static information (i.e., facial expressions and morphological features evaluated by static images).

The body in communication is not static but is rather a dynamic source of psychological information (Cook, 1979; Oberzaucher and Grammer, 2008; Witkower and Tracy, 2019; Hirai and Senju, 2020; Troje and Chang, 2023). Therefore, to understand physical attractiveness and how it is perceived in the real world, the attractiveness perception mechanisms in human movements must be clarified. Walking is the most fundamental human movement and mediates walkers’ socially relevant information such as gender (Smith et al., 2002). In another study, we revealed biomechanical strategies used by female walkers to express their gait attractiveness (Tanabe et al., 2023). We also built statistical models demonstrating the causal relation between gait parameters and gait attractiveness (Tanabe and Yamamoto, 2023), revealing that morphological characteristics and dynamic gait parameters including cadence, horizontal shaking of the head, and taking back of the arms influence one’s perception of attractiveness. These studies suggested that as the observer perceives attractiveness, they explore and process gait parameters as visual information and make judgments of attractiveness accordingly. However, the visual search (i.e., gaze behavior) in such a process remains unknown.

Physical attractiveness is an important factor in sexual selection (Clarkson et al., 2020; Rosenthal and Ryan, 2022; DuVal et al., 2023). Sexual selection is driven by two distinct forces, namely, intrasexual selection (i.e., sexual competition between individuals of the same sex) and intersexual selection (i.e., mate choice; Darwin, 1871). Darwin’s original description of sexual selection stated that it is driven by male competition over access to females. In intrasexual selection, males usually compete with each other for mates, and in intersexual selection, females usually select males. Moreover, in human mate selection, women’s visual processing of men’s bodies changes depending on their menstrual cycle (i.e., women’s visual attention is biased toward the upper region of men’s bodies during periods of high fertility; Garza et al., 2017; Garza and Byrd-Craven, 2019). Although these sex biases are important mechanisms of sexual selection (Janicke et al., 2016), there are also many cases of females competing for access to males, representing a reversal of the sex roles advocated by Darwin (Schlupp, 2021). Human mate selection mechanisms are more complex because they include sociocultural factors (Buss, 1985; Buss and Barnes, 1986); however, physical attractiveness is an important feature of women that is preferred by men (Elder, 1969; Buss, 1985). Even today, physical attractiveness is an important criterion for mate selection by men (Puts, 2010). Furthermore, men with higher status and income tend to marry more physically attractive women (Buss and Schmitt, 2019). In this context, unlike men, who attempt to eliminate male competitors through force, women compete with other women by attempting to attract men (Puts, 2016). In this study, we investigated what bodily cues are used by women to signal physical attractiveness. Notably, we focus on how men evaluate women’s physical attractiveness (which can affect mate selection) and how women evaluate the attractiveness of fellow female competitors, enabling us to obtain insights into the visual cues employed to signal female physical attractiveness and their functions in the contexts of intersexual selection by men and intrasexual competition among women.

Researchers have observed gender differences in the process of perceiving physical attractiveness. For example, when observing both men’s and women’s body images, male observers pay faster and longer attention to women’s chests, whereas female observers pay faster attention to men’s legs (Hewig et al., 2008). Moreover, compared with women, men pay more attention to women’s breasts and heads (Garza et al., 2016). Pazhoohi et al. (2019) have also shown that a gender difference exists in the attraction to shoulder-to-hip ratio (SHR), with men paying more attention to SHR. The context of these gender differences in the attractiveness perception process reflects an evolutionary psychological adaptation (i.e., attractiveness evaluation allows for the avoidance of bad genes in one’s mating strategy). For example, a man’s waist–hip ratio is important for women when assessing a man’s attractiveness (Singh, 1995). Meanwhile, women’s fertility is indicated not only by their waist–hip ratio but also their breast size, which men consider important when evaluating a woman’s attractiveness (Singh, 1993; Singh and Young, 1995). These gender differences in body parts that reflect reproductive ability have led researchers to presume differences in body parts focused on by both genders (i.e., male observers pay more attention to the chest region) when scanning human bodies (Johnson and Tassinary, 2005). Such gender differences in the physical attractiveness perception process should be explained in not only the observation of static body images but also the perception of body movements.

Using data from walking videos, this study sought to clarify (1) gender differences in gaze behavior in the gait attractiveness perception process and (2) the relation between such gender differences and gait attractiveness evaluation models, that is, whether these models reflect gender differences in gaze behavior, which was examined by qualitatively comparing standardized estimates of the models for each gender. We hypothesize that male observers pay more attention to walkers’ trunk area and place greater weight on gait parameters associated with trunk movements in the gait attractiveness evaluation model. This research is expected to deepen our knowledge of the perceptual process of the attractiveness of human movements.

## 2 Methods

All research procedures adhered to the Declaration of Helsinki and were approved by Nagoya University’s Ethics Committee. All participants provided written informed consent to participate and for us to publish case details. Informed consent was secured throughout the study via dialog between researchers and participants.

### 2.1 Experiment for creating gait animation

The participants comprised seven professional runway models (42.4 ± 7.0 years; 170.6 ± 3.7 cm; 55.6 ± 3.4 kg) and 10 nonmodels (34.0 ± 7.2 years; 162.0 ± 5.4 cm; 54.7 ± 7.7 kg) who walked on a treadmill barefoot or wearing high heels (two trials for each condition) at a speed of 1.0 m/s. Using a three-dimensional (3D) optical motion capture system (OptiTrack V100—R2; NaturalPoint, Corvallis, OR) with a sampling frequency of 100 Hz, we obtained the positions of 18 feature points during gait: top of the head, ears, acromions, elbows (calculated as the midpoint between the humerus-medial epicondyle and the humerus-lateral epicondyle), wrists, upper margin of the sternum, sternum-xiphoid process, the ribs’ lowest edge, C7 vertebra, T8 vertebra, T12 vertebra, anterior superior iliac spine, posterior superior iliac spine, greater trochanter, lateral and medial knee joint space, malleolus lateralis and medialis, toe and calcaneus, and bottom of the heel for the high-heel condition.

We produced gait animations using motion capture data for the subsequent attractiveness evaluation experiment. Rather than actual walking scenes, the observers were presented with these gait animations to avoid being influenced by information obtained from the skin, such as the walkers’ age and fat/muscle condition. To create these animations, we passed time series data for joint center coordinates through a fourth-order Butterworth low-pass filter (cutoff frequency 6 Hz) to create a 30-s gait animation, during which, we rotated the animation’s viewpoint at a constant speed from the walker’s front right to back left (Supplementary Videos 1, 2 are sample animations featuring data from barefoot nonmodels and from runway models wearing high heels, respectively). We produced a total of 68 animations (17 participants, 2 footwear conditions, and 2 trials per condition). We also calculated 3D joint angles for the ankle, knee, hip, lumbosacral joint, thoracolumbar joint, neck, shoulder, and elbow, which were used to investigate motion factors that affect observers’ judgment of attractiveness and femininity. All signal processing for creating gait animations was conducted using Matlab R2021a.

### 2.2 Experiment for impression evaluation and gaze behavior

A total of 30 women (aged 20–59 years; 38.50 ± 13.26 years) and 30 men (aged 24–58 years; 40.70 ± 10.59 years) participated in the gait attractiveness and femininity evaluation. All participants were Japanese, and their sexual orientation was heterosexual. They watched the 30-s walking animations presented on a standard computer monitor (EIZO FlexScan EV2480). To avoid assessment based solely on early attentional acquisition in the first second (Nummenmaa et al., 2012), the participants were instructed to finish watching the videos, which were presented randomly, and to keep their eyes fixed on the animation while it was moving. Within 30 s after each animation stopped, the participants rated attractiveness and femininity on a 7-point Likert scale from 1 (low attractiveness and femininity) to 7 (high attractiveness and femininity). The participants were provided no information about the walkers (e.g., age, sex, or occupation), and they took a break after every 17 evaluations.

We measured the gaze behavior of 33 out of the 60 observers, 17 of whom were women (21–59 years; 38.06 ± 14.21) and 16 of whom were men (24–58 years; 42.25 ± 10.32). The eye-tracking data sample was halved due to issues with the availability of experimental equipment and facilities; it was not because these 27 participants dropped out or were excluded. Subsequently, we adjusted the demographic characteristics of the 33 observers to ensure that the gender and age cohort ratios were uniform, similar to the 60 observers who underwent impression evaluation. Thus, although the sample size was halved, the demographic characteristics of the impression evaluation sample (60 observers) and the eye-tracking sample (33 observers) are considered to be almost identical. We obtained their gaze-tracking data using an eye-tracking software (Tobii Pro Lab, Screen Edition, version 1.181) with a Tobii Pro Nano screen-based eye-tracking camera (Tobii, Danderyd, Sweden) attached to the bottom of the computer monitor. Calibration was performed at nine points, including one at the center of the display. The distance between the eye tracker and the participant was kept within the operating distance, which was 45–85 cm from the eye tracker. For this purpose, the chair position and monitor height were adjusted before the experiment and were not moved during the experiment. The sampling frequency was 60 Hz. We kept monitor brightness and room lighting constant throughout the experiment. As measurement began, we performed calibration using the Tobii Pro Lab software by asking the participants to focus on a small white target traveling across the screen.

### 2.3 Analysis of eye-tracking data

**Figure 1:**
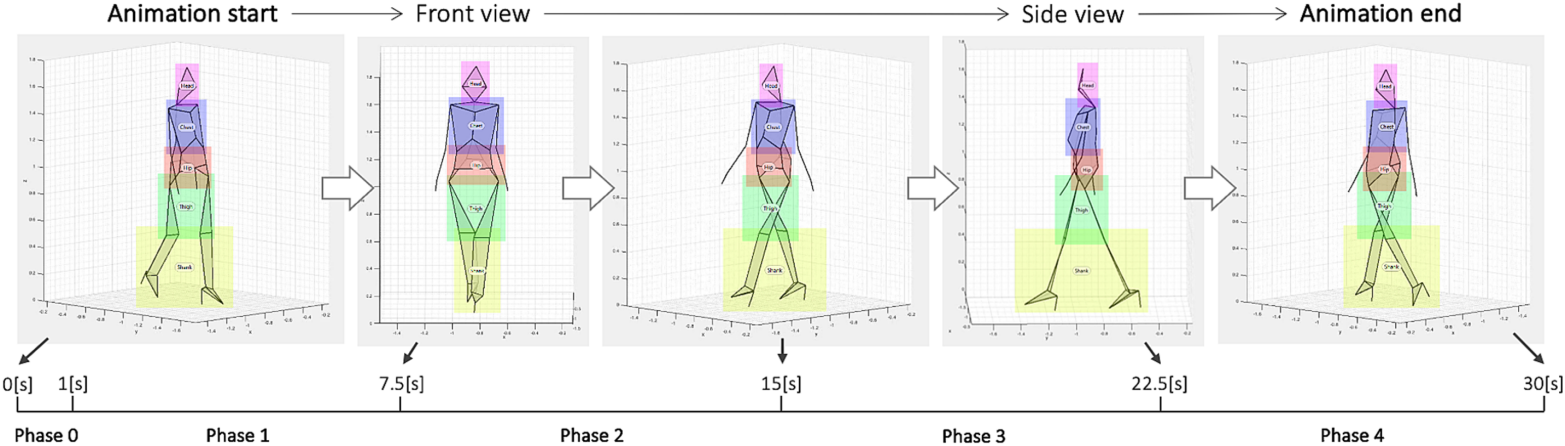
Gait animation flow. During 30 s, the animation’s viewpoint was rotated at a constant speed from the walker’s front right to back left. Data analysis divided the animation into five phases: phase 0–4. Each area of interest (AOI) is displayed in a different color: head in pink, trunk in purple, hip in red, thighs in green, and shanks in yellow. The AOIs were invisible to the observers.

To examine which of the walker’s body parts the observer was observing, we first set five areas of interest (AOIs) for each animation: head, trunk, hip, thigh, and shank. Figure 1 shows each AOI in a different color. We manually configured each AOI using Tobii Pro Lab software AOI tools. All AOIs were the smallest squares that completely covered each body region (head, trunk, hip, and leg including foot). However, because the observation target is moving, a slight margin exists to prevent each part from protruding, and we needed to finely manually adjust the rectangle’s shape along the time series. The software outputs time series information on the following eye movement types: “Fixation,” “Saccade,” “Unclassified,” or “Eyes not found.” We also obtained time series data of AOI hits, which was an array of 0 or 1 (1 if the gaze point is within the AOI, 0 otherwise) for each AOI. In subsequent data analysis, the thigh and shank AOIs gave similar results; hence, we decided to combine them into a single AOI (leg) to show the results.

During walking observation, the observer’s gaze area could be influenced by their gender, preference for the object (i.e., gait attractiveness), and the direction from which the walker is viewed. Therefore, we first divided the 30-s animation into five phases (Figure 1). Phase 0 (0–1 s) is the early attention acquisition period (Nummenmaa et al., 2012). In this study, we distinguished this phase from those that reflect elaborate processing (phases 1–4). Phases 1–4 is a period of 7.5 s, which is 30 s equally divided into four (phase 1 only, 6.5 s excluding phase 0). The most common type of eye-tracking event is eye fixation, which refers to a focused state in which the eyes dwell voluntarily over some time. Therefore, in each phase, we calculated the fixation rate of each AOI in all fixations and used them as an index reflecting the observer’s gaze distribution. Moreover, considering the influence of gait attractiveness on gaze behavior, we first created attractiveness rankings among 68 gait animations and separately pooled fixation rate scores when observing the top and bottom five animations (preference factor: top or bottom). Therefore, to examine gender differences in gaze behavior and clarify the influence of attractiveness, we compared fixation rates based on four factors: gender, preference, phase, and AOI.

All fixation rate data were expressed as mean ± standard deviation (SD). To understand the overall fixation rate trend, we first pooled all phases and performed an analysis of variance (ANOVA) on three factors: gender (male or female), preference (top or bottom), and AOI (head, trunk, hip, leg, or others). Then, focusing on AOIs with relatively high fixation rates and a tendency for gender differences, we conducted ANOVA using three factors: gender (male or female), preference (top or bottom), and phase (0–4). Eta squared was calculated as the effect size with η^2^ of 0.01, 0.06, and 0.14 representing small, medium, and large effects, respectively (Cohen, 1988). The Tukey’s *post hoc* analysis was conducted expecting for the differences in mean fixation rate between elements within each factor. All signal processing for calculating fixation rate was implemented using Matlab R2022b and R2023a.

### 2.4 Structural equation modeling

To examine how gender differences in gaze behavior affect the cognition of gait attractiveness, we constructed gait attractiveness models using structural equation modeling (SEM; Ullman and Bentler, 2012) for each gender. Unlike linear regression models, assuming that predictor variables are fixed or measured without error (Fox, 2015), SEM can combine measurement models and structural (i.e., regression) models into a single overarching model that optimally handles measurement error in predictor variables. The SEM models that represent the causal relationship between the parameters and impression scores of walkers illustrate what information regarding walkers is processed as visual cues by observers and how they perceive gait attractiveness. In another study, we created statistical models by SEM for female gait attractiveness using gait parameters correlated with impression scores as explanatory variables (Tanabe and Yamamoto, 2023). The model comprises four latent variables: physique, trunk silhouette, head silhouette, and health factors. The physique factor consists of static elements that characterize the walkers’ body shape. In our previous study (Tanabe and Yamamoto, 2023), we examined walkers’ body mass index (BMI), height, and weight as explanatory variables contributing to the physique factor. However, only BMI demonstrated a significant correlation with the impression score. Consequently, in the present study, the physique factor is also operationalized based on BMI. The trunk and head silhouettes represent the dynamic contours formed by the alignment of the trunk and head segments during walking. We previously identified lumbar curvature, backward arm swing, forward head tilt, and horizontal head shake as features significantly correlated with impression scores (Tanabe and Yamamoto, 2023). Accordingly, these parameters were included as explanatory variables for the trunk and head silhouette factors. In addition, the health factor includes knee extension and cadence, both of which have been associated with age-related decline in walking speed and walking capacity (Hamrin et al., 1982; Nakamura et al., 1985; Bohannon, 1997), as well as orthopedic conditions such as knee osteoarthritis (Boekesteijn et al., 2022).

In this study, although the hypothesized models to be tested were based on our previous SEM models, the data for each gender were pooled separately. A total of 1,017 and 1,018 data samples were retrieved for male observers for the barefoot and high-heel conditions, respectively (17 walkers × 2 trials × 30 observations), whereas a total of 1,020 data samples were obtained for female observers for both conditions. The following procedures were conducted for the structural analysis of multiple populations (i.e., multi-group SEM for male and female data): (1) assessing model fit for each population, (2) conducting a configuration invariance analysis to determine whether the model structure is consistent across populations, and (3) performing a measurement invariance analysis to verify both structure consistency and the equivalence of parameter estimates across populations. For configuration and measurement invariance testing, four models were specified, and their goodness of fit was assessed: (a) a configuration invariance model with no equality constraints; (b) a weak measurement invariance model with equality constraints on factor loadings; (c) a measurement invariance model with equality constraints on factor loadings and covariances; and (d) a strong measurement invariance model with equality constraints on factor loadings, covariances, and error variances.

We performed SEM using SPSS Amos (IBM SPSS 29.0, Amos version 29) with a visual and intuitive interface. We evaluated the models using the chi-square test (*χ*^2^) and overall fit indices provided by SPSS Amos. Ideally, *χ*^2^ values for a model that fits the data would not be significant (*p* > 0.05). However, because SEM is based on covariances, the chi-square test is highly sensitive to sample size; as sample size increases while degrees of freedom remain constant, the *χ*^2^ value also increases, often leading to a small *p*-value. Thus, “not too much emphasis should be placed on the significance of the *χ*^2^ statistic” (Schermelleh-Engel et al., 2003, p. 12), and model fit should be assessed comprehensively using additional fit indices. We also evaluated model fit according to four popular fit indices: the goodness of fit index (GFI; Jöreskog and Sörbom, 1984; Tanaka and Huba, 1984), the adjusted goodness of fit index (AGFI; Jöreskog and Sörbom, 1984), the comparative fit index (CFI; Bentler, 1990), and the root mean square error of approximation (RMSEA; Browne and Cudeck, 1993). GFI ranges from 0 to 1, with higher values indicating better fit, and a GFI > 0.95 usually indicates good fit relative to the baseline model, whereas a GFI > 0.90 is usually interpreted as acceptable fit (Marsh and Grayson, 1995; Schumacker and Lomax, 2022). AGFI also typically ranges from 0 to 1, with higher values indicating better fit; an AGFI > 0.90 signifies good fit compared with the baseline model, whereas an AGFI > 0.85 is considered acceptable. AGFI is usually smaller than GFI, and AGFI approaches GFI as the target model’s degrees of freedom move toward those of the null model. Further, a CFI > 0.95 is often indicative of good-fitting models (Hu and Bentler, 1999). RMSEA, which estimates a model’s lack of fit and measures noncentrality relative to sample size and degree of freedom, denotes a close-fitting model with values of ≤0.06 (Hu and Bentler, 1999), whereas values >0.10 indicate a poor-fitting model (Browne and Cudeck, 1993). Schermelleh-Engel et al. (2003) provide details regarding these fit measures’ definitions and calculations.

## 3 Results

### 3.1 Gaze analysis during walking observation

**Figure 2:**
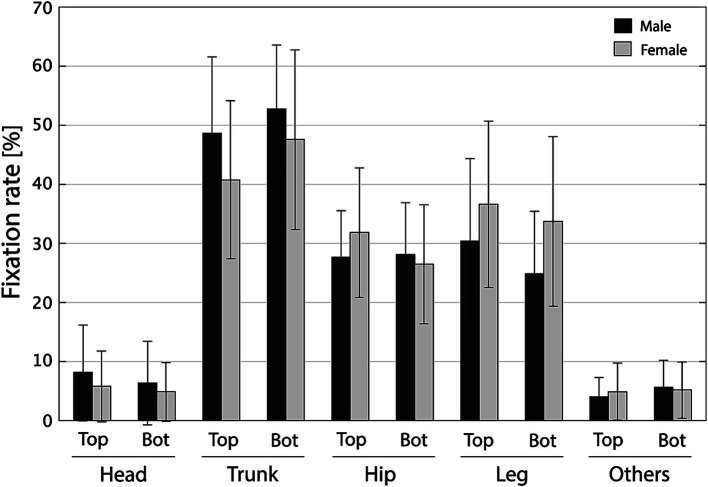
Fixation rate of each area of interest (AOI) (head, trunk, hip, leg, and others). Fixation rates for the top (Top) and bottom (Bot) five attractive animations were pooled separately. The mean fixation rate is shown by observer gender: dark gray for males and light gray for females. Error bars represent standard deviations.

Out of the 33 eye-tracking participants, data from 3 (1 male, 2 females) were excluded as their data could not be successfully obtained because of insufficient calibration accuracy. We first aimed to compare fixation rates between AOIs and captured the overall trend of gender differences in these rates. Therefore, we initially pooled all phases and performed an ANOVA on three factors: AOI (head, trunk, hip, leg, or others), gender (male or female), and preference (top or bottom, reflecting that the animations observed by the observer were in the top or bottom five of the attractiveness rankings among 68 animations). Figure 2 shows the average fixation rate in all phases for each factor (i.e., AOI, preference, and gender). Fixation rates for the top five (Top) and bottom five (Bot) attractive animations were pooled separately. The mean ± SD of fixation rate is shown by observer gender, with dark gray for men and light gray for women. Three-way ANOVA revealed a main effect for the AOI with a large effect size: *F*_(4,280)_ = 190.96, *p* < 0.001, η^2^ = 0.713. We also observed an AOI × gender interaction albeit with a small effect size: *F*_(4,280)_ = 3.86, *p* = 0.0045, η^2^ = 0.0144. No other main effects or interactions were found [gender: *F*_(1,280)_ = 0.0108, *p* = 0.917, η^2^ < 0.0001; preference: *F*_(1,280)_ = 0.0692, *p* = 0.793, η^2^ < 0.0001; AOI × preference: *F*_(4,280)_ = 2.08, *p* = 0.0842, η^2^ = 0.00775; gender × preference: *F*_(1,280)_ = 0.00436, *p* = 0.947, η^2^ < 0.0001; AOI × gender × preference: *F*_(4,280)_ = 0.463, *p* = 0.763, η^2^ = 0.00173]. Furthermore, regarding the interaction between AOI and gender, the fixation rate for trunks tended to be higher among men while that for legs tended to be higher among women. Subsequent multiple comparisons with Tukey’s honestly significant difference (HSD) method showed fixation rate at its peak in the trunk area (approximately 40–50%; *p* < 0.001 in all pairs), followed by the hip (*p* < 0.001 in comparison with the head and others AOI) and leg (*p* < 0.001 in comparison with the head and others AOI) areas (around 30%; *p* = 0.5256 for the comparison between hip and leg AOIs) and then the head and other areas (less than 10%; *p* = 0.9449 for the comparison between head and others AOIs). According to the above statistical analysis and Figure 2, the observers mainly distributed their gaze in the trunk, hip, and leg, and gender differences were observed in the AOIs of the trunk and leg. Therefore, the subsequent analysis focused on the AOIs of the trunk and the leg and performed a three-way ANOVA with factors of gender, preference, and phase for each AOI.

**Figure 3:**
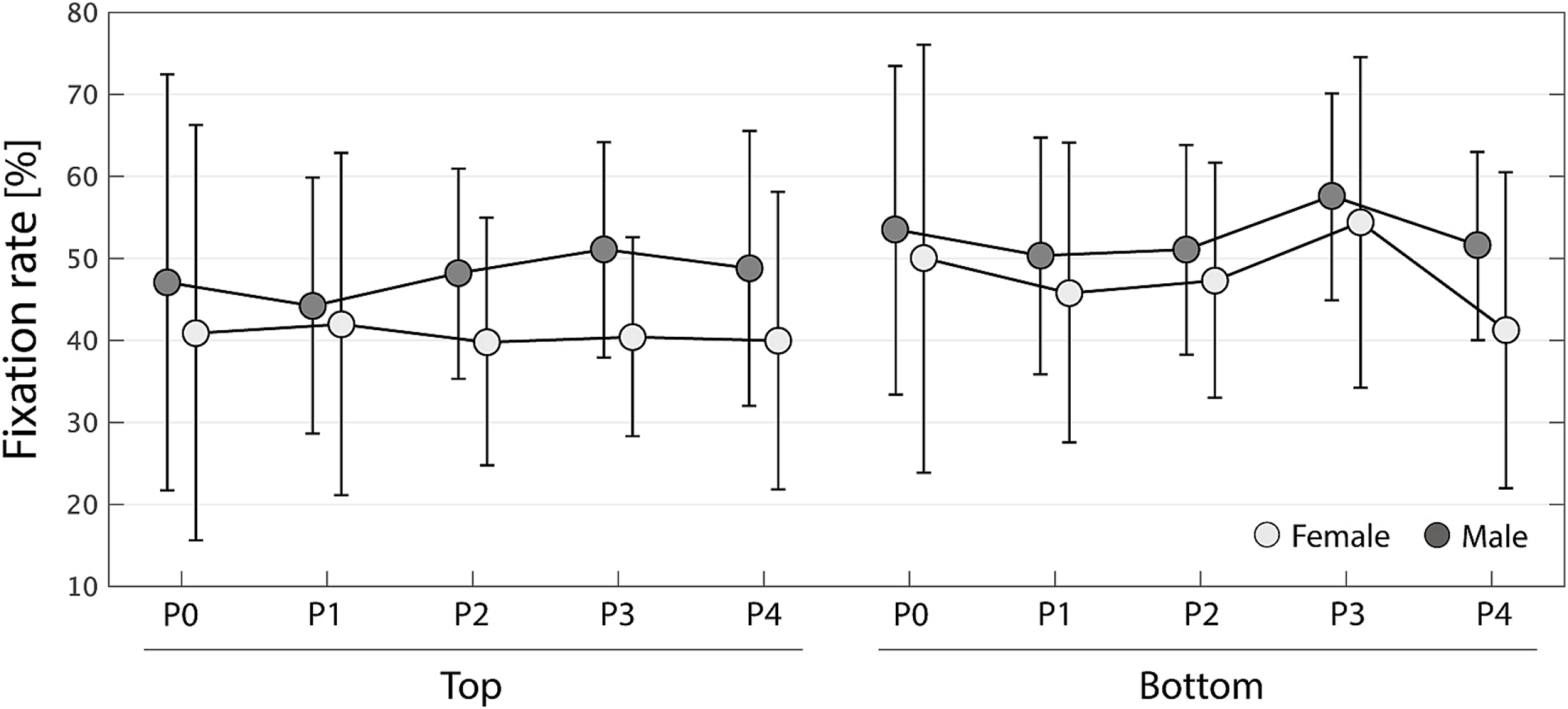
Changes in fixation rate of the trunk area of interest for each gender (females in light gray and males in dark gray). P0 to P4 represent phases 0–4. Error bars represent standard deviations.

Figure 3 shows changes in the fixation rate of the trunk for each gender and preference in each phase. The data show means ± SD. Female and male data are shown in light and dark gray, respectively, and P0–P4 correspond to phases 0–4, respectively. The three-way ANOVA showed a main effect for gender and preference with small to medium effect size: for gender, *F*_(1,280)_ = 8.90, *p* = 0.00310, η^2^ = 0.0291, and for preference, *F*_(1,280)_ = 8.55, *p* = 0.00374, η^2^ = 0.0280. There were no other main effect [phase: *F*_(4,280)_ = 0.942, *p* = 0.440, η^2^ = 0.0123] and interactions [gender × preference: *F*_(1,280)_ = 0.280, *p* = 0.597, η^2^ < 0.001; gender × phase: *F*_(4,280)_ = 0.254, *p* = 0.907, η^2^ = 0.00332; preference × phase: *F*_(4,280)_ = 0.449, *p* = 0.773, η^2^ = 0.00588; gender × preference × phase: *F*_(4,280)_ = 0.200, *p* = 0.938, η^2^ = 0.00261]. Afterward, multiple comparisons with Tukey’s HSD method revealed that the fixation rate of the trunk was higher for male observers (*p* < 0.001) and bottom preference (*p* = 0.0063) compared with female observers and top preference, respectively. Figure 3 illustrates the tendency that when observing a gait with low attractiveness (bottom), the observer’s trunk fixation rate is around 50% (slightly higher for males) regardless of their gender; meanwhile, when observing a gait with high attractiveness (top), the trunk fixation rate is around 50 and 40% for male and female observers, respectively.

**Figure 4:**
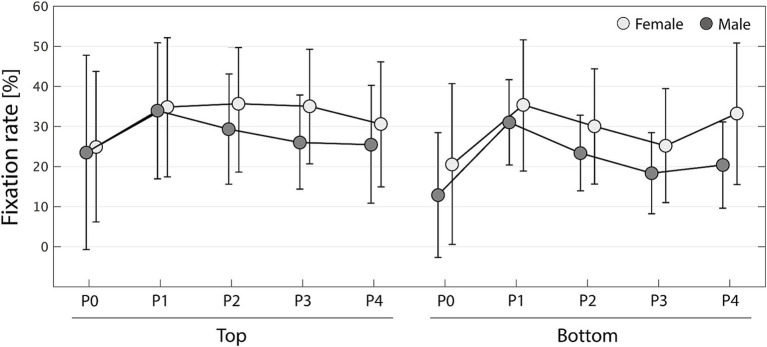
Changes in fixation rate of the leg area of interest for each gender (females in light gray and males in dark gray). P0 to P4 represent phases 0–4. Error bars represent standard deviations.

Similarly, Figure 4 shows how the leg fixation rate transitions for each gender and preference in each phase. The data show means ± SD. Female and male data are shown in light and dark gray, respectively, and P0–P4 correspond to phases 0–4, respectively. The three-way ANOVA showed main effects of gender, preference, and phase factors: for gender, *F*_(1,280)_ = 11.7, *p* < 0.001, η^2^ = 0.0354; for preference, *F*_(1,280)_ = 7.46, *p* = 0.00672, η^2^ = 0.0226; and for phase, *F*_(4,280)_ = 5.96, *p* < 0.001, η^2^ = 0.0725. All interactions were not obtained [gender × preference: *F*_(1,280)_ = 0.752, *p* = 0.387, η^2^ = 0.00228; gender × phase: *F*_(4,280)_ = 0.412, *p* = 0.800, η^2^ = 0.00501; preference × phase: *F*_(4,280)_ = 0.772, *p* = 0.544, η^2^ = 0.00937; gender × preference × phase: *F*_(4,280)_ = 0.260, *p* = 0.903, η^2^ = 0.00316]. According to multiple comparisons with Tukey’s HSD method, leg fixation rate was significantly higher for women than for men (*p* < 0.001), for top preference than for bottom (*p* = 0.0063), and for phases 1 and 2 than for phase 0 (*p* < 0.001 and *p* = 0.0108, respectively). Figure 4 shows that during the early attentional phase (phase 0), observers pay relatively little attention to the leg, but in the subsequent observation (phase 1), the proportion of fixations tends to increase. Additionally, after phase 1, the leg fixation rate decreases for male observers, especially when observing the lower-attractiveness gait (bottom). Meanwhile, for women, leg fixation remains above 30% when observing a highly attractive gait (top), and even when observing a gait with low attractiveness (bottom), it decreases in phases 2 and 3 but recovers to over 30% in phase 4.

### 3.2 Gender differences in gait attractiveness evaluation models

This study further examined gender differences in cognitive models of gait attractiveness. In another study (Tanabe and Yamamoto, 2023), we revealed four models for female gait attractiveness (two for barefoot walking and two for high-heel walking) using women’s gait parameters as explanatory variables. In this study, we performed SEM for each observer gender based on each model. By comparing the standardized estimates of each path between genders, we examined gender difference trends in gait attractiveness evaluation models.

**Figure 5:**
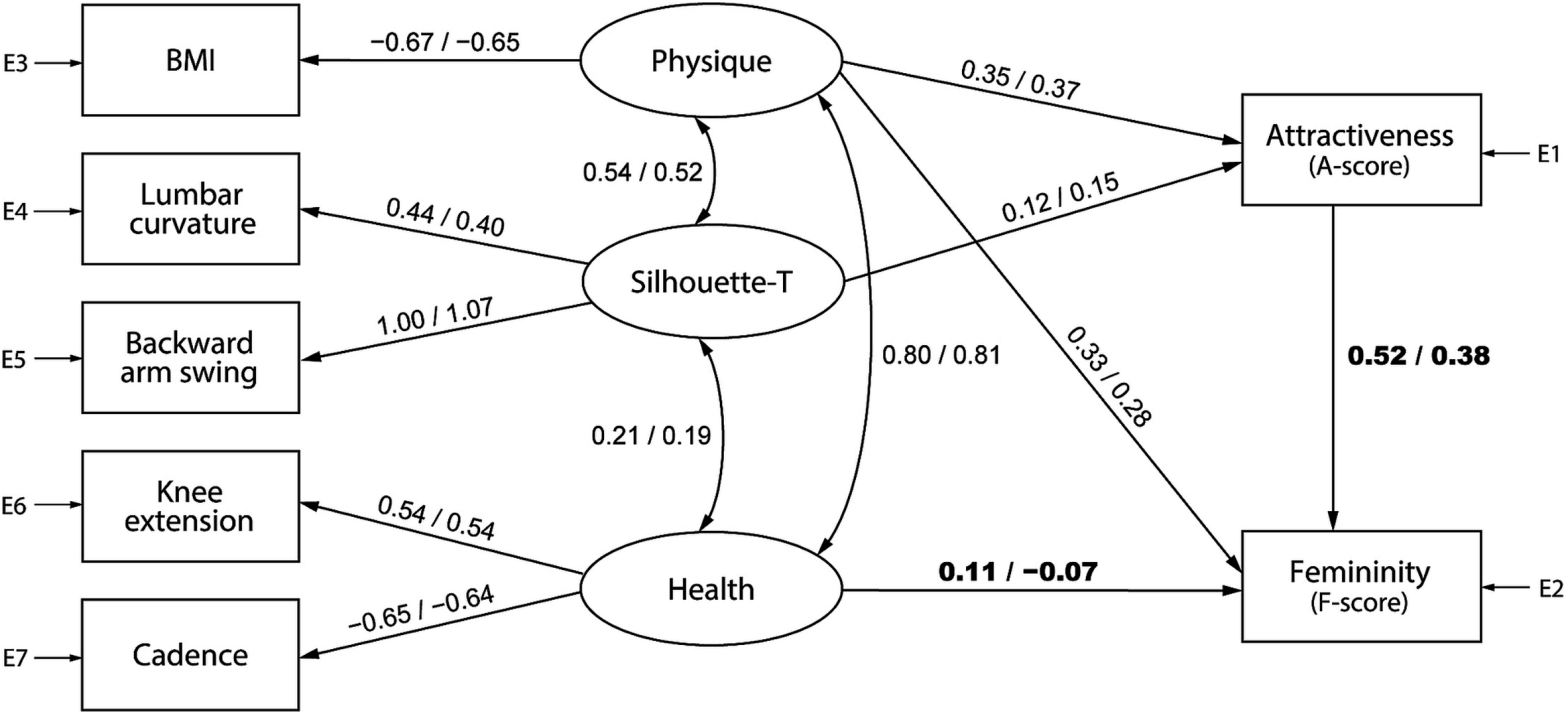
A barefoot gait attractiveness model that uses physique, trunk silhouette (Silhouette-T), and health factors as evaluation criteria. The numbers on each path indicate standardized estimates, with values from the model for female observers on the left and male observers on the right.

**Figure 6:**
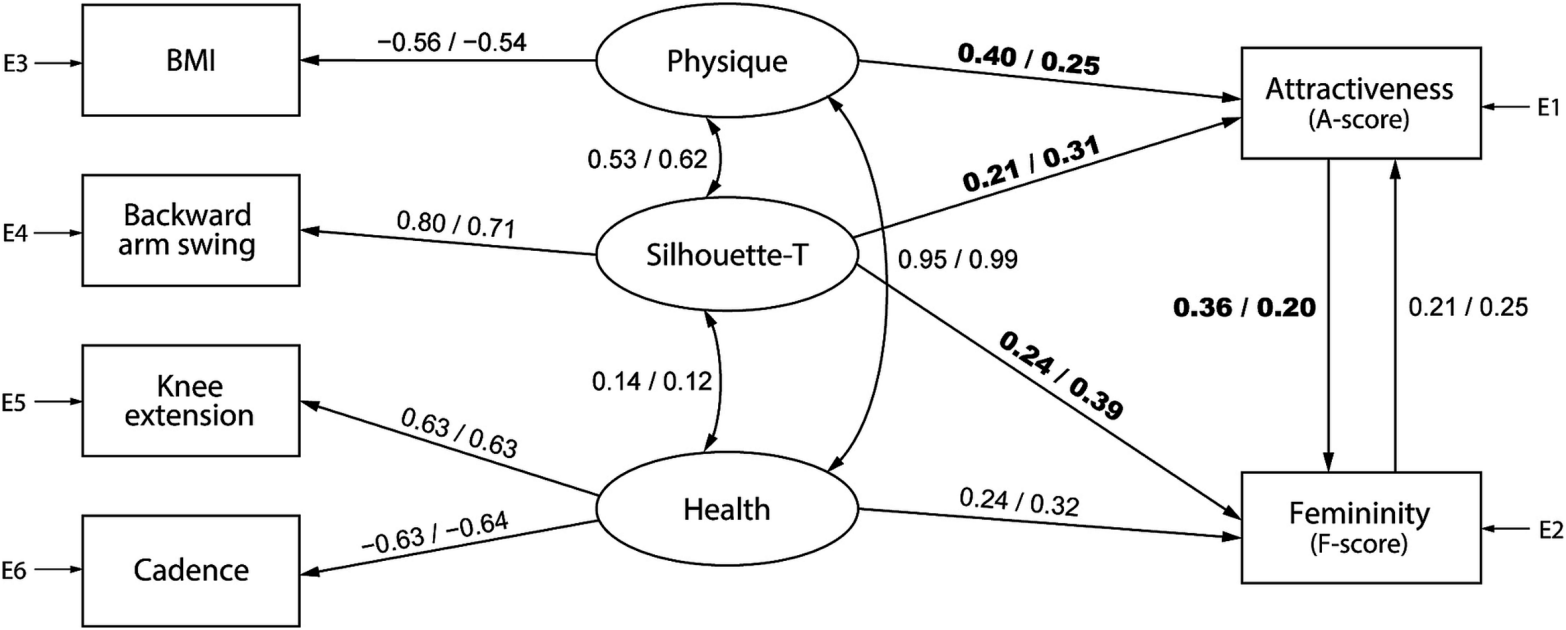
A high-heel gait attractiveness model that uses physique, trunk silhouette (Silhouette-T), and health factors as evaluation criteria. The numbers on each path indicate standardized estimates, with values from the model for female observers on the left and male observers on the right.

Figures 5, 6 represent gait attractiveness models comprising physique, trunk silhouette (Silhouette-T), and health factors for barefoot and high-heel walking, respectively. For each model, the feature values calculated from gait data are placed on the left side, the observer’s impressions (i.e., gait attractiveness and femininity) are placed on the right side, and the causal relations between them are expressed as paths. SEM was performed by separately pooling data of each gender of observers. Thus, the numbers on each path indicate standardized estimates, with values from the model for female and male observers on the left and right, respectively. In the barefoot walking model (Figure 5), although the *p*-value of the chi-square test was less than 0.05, the fit indices were acceptable: for female observers, df = 8, *χ*^2^ = 52.7, *p* < 0.001, GFI = 0.985, AGFI = 0.948, CFI = 0.929, RMSEA = 0.074, and for male observers, df = 8, *χ*^2^ = 31.5, *p* < 0.001, GFI = 0.991, AGFI = 0.969, CFI = 0.958, RMSEA = 0.054. Similar results were observed for the heel walking model (Figure 6): for female observers, df = 3, *χ*^2^ = 24.3, *p* < 0.001, GFI = 0.992, AGFI = 0.944, CFI = 0.959, RMSEA = 0.083, and for male observers, df = 3, *χ*^2^ = 31.0, *p* < 0.001, GFI = 0.990, AGFI = 0.929, CFI = 0.946, RMSEA = 0.096. Of the two standardized estimates listed above each path, the left and right ones show the results when using the female and male evaluation scores, respectively.

Tables 1, 2 present the results of the configuration invariance and measurement invariance analysis for the models shown in Figures 5, 6, respectively. For both models, the configuration invariance model demonstrated an acceptable fit, indicating that the model structure is likely consistent across genders. In addition, all measurement invariance models exhibited acceptable fit, suggesting no significant differences in the estimated values between men and women. However, the AIC value was lower for the group-specific models, implying that separate models for men and women can better predict the objective variables. Although this is a qualitative analysis, cases where the gender difference in the standardized estimates was greater than 0.1 are shown in bold. In the barefoot walking model (Figure 5), the path weights from the health factor to femininity (female: 0.11, male: −0.07) and from attractiveness to femininity (female: 0.52, male: 0.38) tended to be greater for female observers. Additionally, for the heel walking model (Figure 6), the path weights from the physique factor to attractiveness (female: 0.40, male: 0.25) and from attractiveness to femininity (female: 0.36, male: 0.20) were higher for female observers, and the path weights from the trunk silhouette factor to attractiveness (female: 0.21, male: 0.31) or femininity (female: 0.24, male: 0.39) were larger for male observers. These gender differences in standardized estimates may affect the better AIC values of the group-specific models.

**Table 1:** Fit indices for the barefoot gait attractiveness model (Figure 5), including group-specific models (male and female), the configuration invariance model (CI model), and measurement invariance models (MI models).

Models		*χ* ^2^	df	*p*	GFI	AGFI	CFI	RMSEA	AIC
Group-specific models	Female	52.67	8	<0.001	0.985	0.948	0.929	0.074	92.67
	Male	31.47	8	<0.001	0.991	0.969	0.958	0.054	71.47
CI model		85.46	16	<0.001	0.988	0.959	0.974	0.046	165.45
Weak MI model		105.94	23	<0.001	0.986	0.965	0.969	0.042	171.94
MI model		107.21	29	<0.001	0.985	0.972	0.970	0.036	161.21
Strong MI model		112.79	36	<0.001	0.985	0.976	0.971	0.032	152.79

**Table 2:** Fit indices for the heel gait attractiveness model (Figure 6), including group-specific models (male and female), the configuration invariance model (CI model), and measurement invariance models (MI models).

Models		*χ* ^2^	df	*p*	GFI	AGFI	CFI	RMSEA	AIC
Group-specific models	Female	24.31	3	<0.001	0.992	0.944	0.959	0.083	66.31
	Male	30.99	3	<0.001	0.990	0.929	0.946	0.096	72.99
CI model		55.29	6	<0.001	0.991	0.937	0.952	0.064	127.29
Weak MI model		65.76	13	<0.001	0.989	0.965	0.949	0.045	125.76
MI model		67.76	19	<0.001	0.989	0.975	0.953	0.036	115.76
Strong MI model		70.82	25	<0.001	0.988	0.981	0.956	0.030	106.82

**Figure 7:**
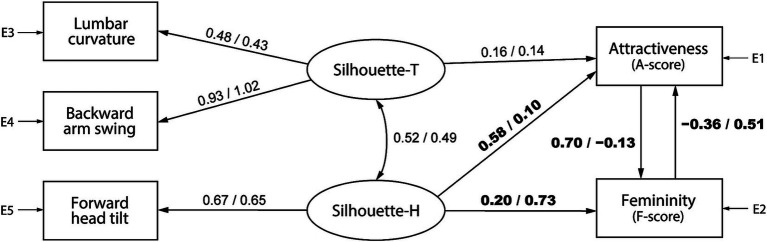
A barefoot gait attractiveness model that uses trunk and head silhouette (Silhouette-T and Silhouette-H, respectively) factors as evaluation criteria. The numbers on each path indicate standardized estimates, with values from the model for female observers on the left and male observers on the right.

**Figure 8:**
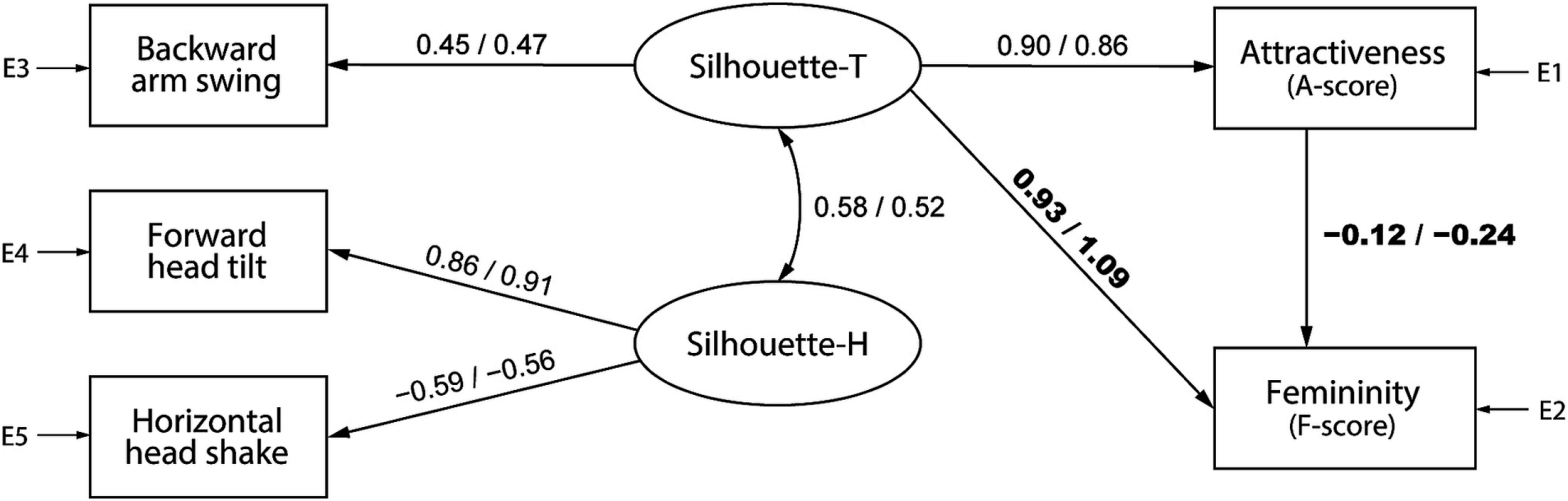
A high-heel gait attractiveness model that uses trunk and head silhouette (Silhouette-T and Silhouette-H, respectively) factors as evaluation criteria. The numbers on each path indicate standardized estimates, with values from the model for female observers on the left and male observers on the right.

Figures 7, 8 illustrate the attractiveness models for barefoot and high-heeled walking, comprising trunk and head silhouettes (Silhouette-T and Silhouette-H, respectively), and show the SEM results by pooling data according to the observer’s gender. The numbers on each path indicate standardized estimates, with values from the model for female and male observers on the left and right, respectively. In the barefoot walking model (Figure 7), although the *p*-value of the chi-square test was below 0.05, the fit indices were acceptable: for female observers, df = 2, *χ*^2^ = 24.2, *p* < 0.001, GFI = 0.990, AGFI = 0.929, CFI = 0.954, RMSEA = 0.104, and for male observers, df = 2, *χ*^2^ = 12.4, *p* = 0.002, GFI = 0.995, AGFI = 0.963, CFI = 0.977, RMSEA = 0.072. Similar results were found for the heel walking model (Figure 8): for female observers, df = 3, *χ*^2^ = 12.6, *p* = 0.006, GFI = 0.995, AGFI = 0.975, CFI = 0.981, RMSEA = 0.056, and for male observers, df = 3, *χ*^2^ = 22.9, *p* < 0.001, GFI = 0.991, AGFI = 0.955, CFI = 0.961, RMSEA = 0.081.

The results of the configuration variance and measurement invariance analysis are presented in Tables 3, 4 for the models shown in Figures 7, 8, respectively. For both models, the configuration invariance model demonstrated an acceptable fit, indicating that the model structure is likely consistent across genders. In addition, all measurement invariance models exhibited acceptable fit, suggesting no significant differences in the estimated values between men and women. However, the AIC value was lower for the group-specific models, implying that separate models for men and women can better predict the objective variables. A qualitative examination of gender differences in standardized estimates showed that in the barefoot walking model (Figure 7), the path weights from the head silhouette factor to attractiveness (female: 0.58, male: 0.10) and from attractiveness to femininity (female: 0.70, male: −0.13) tended to be larger for female observers; moreover, the path weights from head silhouette to femininity (female: 0.20, male: 0.73) and from femininity to attractiveness (female: −0.36, male: 0.51) tended to be larger for male observers. In addition, for the heel walking model (Figure 8), the effect of trunk silhouette on femininity (female: 0.93, male: 1.09) and the negative effect of attractiveness on femininity (female: −0.12, male: −0.24) tended to be larger for male observers. These gender differences in standardized estimates may affect the better AIC values of the group-specific models.

**Table 3:** Fit indices for the barefoot gait attractiveness model (Figure 7), including group-specific models (male and female), the configuration invariance model (CI model), and measurement invariance models (MI models).

Models		*χ* ^2^	df	*p*	GFI	AGFI	CFI	RMSEA	AIC
Group-specific models	Female	24.25	2	<0.001	0.990	0.929	0.954	0.104	50.25
	Male	12.44	2	0.002	0.995	0.963	0.977	0.072	38.43
CI model		36.68	4	<0.001	0.993	0.946	0.965	0.063	88.68
Weak MI model		43.61	9	<0.001	0.991	0.971	0.963	0.043	85.61
MI model		48.79	12	<0.001	0.990	0.976	0.960	0.039	84.79
Strong MI model		63.91	17	<0.001	0.987	0.978	0.949	0.037	89.91

**Table 4:** Fit indices for the heel gait attractiveness model (Figure 8), including group-specific models (male and female), the configuration invariance model (CI model), and measurement invariance models (MI models).

Models		*χ* ^2^	df	*p*	GFI	AGFI	CFI	RMSEA	AIC
Group-specific models	Female	12.60	3	0.006	0.995	0.975	0.981	0.056	36.60
	Male	22.86	3	<0.001	0.991	0.955	0.961	0.081	46.86
CI model		35.46	6	<0.001	0.993	0.965	0.971	0.049	83.46
Weak MI model		40.62	10	<0.001	0.992	0.976	0.970	0.039	80.62
MI model		41.46	13	<0.001	0.992	0.981	0.972	0.033	75.46
Strong MI model		44.87	18	<0.001	0.991	0.985	0.974	0.027	68.87

## 4 Discussion

This study examined gender differences in the relation between observers’ attractiveness evaluation of walking movements and attentional processes. To this end, we created animations from motion capture data of a woman walking and tracked the observers’ eye movements as they watched the animations and provided their impressions (attractiveness and femininity). When we calculated the fixation rate for each of the five set AOIs in the walking animation (head, trunk, hip, leg, or others), we observed a tendency for gender differences in fixation rate for the trunk and leg areas. Therefore, we focused on the trunk and leg AOIs and compared fixation rates according to observer gender, gait preference (attractive vs. less attractive), and animation phase. Furthermore, we used SEM to examine whether gender differences in observers’ gaze behavior are present in the gait attractiveness evaluation model.

According to eye-tracking analysis results, the highest fixation rate during gaze observation was for the trunk AOI (approximately 50%) followed by the leg AOI (approximately 30%; Figure 2). ANOVA with gender, preference, and phase factors resulted in the main effects of gender and preference for both the trunk and leg AOIs. The subsequent *post hoc* analysis revealed that the fixation rate on the trunk was higher for male than for female observers, and for gaits with low preference (Figure 3); meanwhile, the fixation rate on the legs was higher for female than for male observers, and for gaits with higher preference (Figure 4). These results indicated that, on one hand, male observers focused on the trunk at a higher rate compared with female observers; on the other hand, female observers tended to fixate more on the legs. Although the fixation rate of female observers remained at 30–40%, that of male observers tended to decrease over time. The results also suggested that observers of a highly attractive gait would allocate more attention to the legs. Hewig et al.’s (2008) analysis of gaze behavior when observing human body images showed that male observers spent more time looking at women’s chests, and female observers focused their attention more quickly on men’s legs. Although all objects of observation in this study were women’s gait, this study revealed that when observing walking movements, men pay more attention to the trunk, including the chest, while women focus more on the legs. Further, gait attractiveness may shift observers’ gaze from the trunk to the legs, suggesting a hierarchical process of gait attractiveness perception where attractiveness is first determined by observing the trunk, and once the attractiveness criteria are met, the process moves toward observing the legs. In facial attractiveness perception, observers fixate longer on highly attractive faces (Leder et al., 2016). Thus, the results may be interpreted as follows: in gait attractiveness perception, low-attractiveness gaits are evaluated by observing mostly the trunk whereas high-attractiveness gaits require prolonged observation, during which attention shifts from the walkers’ trunk to their legs. This study set a uniform observation time for all subjects, so the point at which they judged attractiveness was unclear. Further research would help determine the time characteristics of attractiveness perception in walking.

We further conducted SEM for each observer’s gender based on the gait attractiveness evaluation model in another study (Tanabe and Yamamoto, 2023). This enabled us to confirm a causal relationship between gait parameters and impression scores and clarify gender differences in the perception processes for assessing gait attractiveness. Although no statistically significant gender differences were detected in the models (Tables 1–4), by qualitatively comparing the standardized estimates of the models for men and women, we found that the gait attractiveness evaluation models tend to reflect the abovementioned gender differences in gaze behavior. In the barefoot walking model (Figure 5), the path weights from the health factor to femininity tended to be greater for female observers. As the health factor, which consists of knee extension and cadence, could be scanned from one’s observation of the legs, this is consistent with the higher leg fixation rate found among women. In addition, the path weights from the physique factor to attractiveness tended to be greater for female observers in the heel walking model (Figure 6). Estimating a walker’s BMI would require the observation of the whole body; therefore, the finding that female observers disperse their gaze from the trunk to the legs does not contradict the finding that BMI has a greater influence on attractiveness evaluations for female observers. Meanwhile, for heel walking observation, the path weights from the trunk silhouette factor to attractiveness (Figure 6) and femininity (Figures 6, 8) tended to be larger for male observers. The attractiveness evaluation model would reflect male observers’ higher fixation rate of the trunk compared with female observers (Figure 3). Interestingly, the head silhouette factor, which consists of forward head tilt, affected femininity evaluation more for male observers and attractiveness evaluation for female observers (Figure 7). This could indicate gender differences in the causal relation between attractiveness and femininity; that is, for female observers, forward head tilt first elicits attractiveness perception, leading to femininity ratings, whereas for male observers, femininity ratings are made first, and then attractiveness perceptions are elicited. This suggests gender differences in the hierarchy of gait attractiveness and femininity evaluation processes.

Two distinct forces impact sexual selection among animals, including humans, namely, intrasexual selection (i.e., sexual struggle between members of the same sex) and intersexual selection (i.e., mate choice; Darwin, 1871). Moreover, physical attractiveness is a crucial factor affecting the dynamics of sexual selection (Clarkson et al., 2020; Rosenthal and Ryan, 2022; DuVal et al., 2023). In humans, female physical attractiveness is an important criterion in male mate selection (Elder, 1969; Buss, 1985; Puts, 2010; Buss and Schmitt, 2019), and women compete with one another by attempting to attract males rather than by fighting for dominance (Puts, 2016). In this study, we examined how the physical attractiveness of women is perceived through physical bodily cues, focusing on men’s evaluations of the attractiveness of women (as potential mates) and women’s evaluations of the attractiveness of other women (as potential rivals). In the context of intersexual selection by men, our findings show that men focus their observations on the trunks of walking females (with a fixation rate of approximately 50%; Figure 3). Furthermore, in the process of assessing gait attractiveness, men tended to place more importance on the trunk silhouette (Figure 6). This finding is consistent with previous research demonstrating that compared with women, men pay more visual attention to women’s breasts and heads (Garza et al., 2016). Thus, the evolutionary view that reproductive regions of the female body are important for female attractiveness was supported in our observations of male mate choice behaviors.

Meanwhile, from the perspective of intrasexual selection, women’s evaluation of other women’s attractiveness can be understood as assessments of potential competitors. This study revealed that compared with male observers, female observers allocate more visual attention to the legs and that attractive (i.e., higher preference) gaits attract increased attention to the legs (Figure 4). In addition, female observers tended to place increased importance on BMI, knee extension, cadence, and head silhouette, requiring a full-body observation that includes the legs, when evaluating gait attractiveness (Figures 5–7). The BMI of the walkers in this study was 20.01 ± 1.89 (min. 17.02, max. 24.07), aligning with a range from underweight to normal weight (Nuttall, 2015); however, the criterion was that the lower the BMI, the more attractive the individual (Figures 5, 6). Considering that the BMI and fat mass for women preferred by men is below the healthy standard (Brierley et al., 2016) and that the mass media tends to use underweight bodies as icons of beauty, thus creating gender norms that affect girls (Groesz et al., 2002; Hawkins et al., 2004; Grabe et al., 2008), women may evaluate the attractiveness of other women based on the dominant beauty standards of their sociocultural environment (Nicholson, 1994). Moreover, considering that head silhouettes can function as nonverbal messages reflecting a walker’s defensive attitude (Bernhardt, 2022) and emotions (Gross et al., 2012; Venture et al., 2014), the walker’s psychological state may be a factor considered when evaluating the attractiveness of potential competitors. According to our knowledge, our findings regarding the mechanism of attractiveness perception during the process of female intrasexual selection (i.e., evaluation of competitors) constitutes a novel finding, and further research is needed on the sociocultural norms that affect the evaluation of physical attractiveness.

This study has several limitations. First, the data used to generate walking animations were limited to female gaits. Moreover, as all participants were Japanese, the results may be specific to Japanese culture. To more comprehensively clarify the mechanisms underlying the perception and evaluation of gait attractiveness more comprehensively, future studies must consider men’s gaits and individuals from diverse cultural backgrounds. Furthermore, due to issues with the experimental environment and equipment availability, gaze data was limited to 33 participants. A power analysis using G*power 3.1 determined that a sample size of at least 80 is necessary to achieve a power of 0.80 for 3-way ANOVA (2 sex × 2 preference × 5 phase) with a large effect size (η^2^ of 0.14). Therefore, although we identified gender differences in gaze behavior related to attractiveness perception as expected, increasing the sample size could change the interpretation of items where no significant differences were found, such as the interrelationships between AOI, gender, preference, and phase. By increasing the sample size through additional experiments, reducing the estimation error and avoiding missing significant differences due to low statistical power may be possible.

In conclusion, when observing gaits and perceiving their attractiveness, male observers focused more on the walkers’ trunk, whereas female observers distributed their attention to the trunk and legs. This gender difference in gaze behavior was also observed in the gait attractiveness evaluation model. Gait parameters requiring observation of the legs and the whole body for female observers and parameters related to the trunk silhouette for male observers would have a greater influence on gait attractiveness. These results indicate gender differences in gaze behavior when evaluating and perceiving attractiveness of human movements and suggest that such differences may reflect each gender’s evaluation model. The findings help strengthen one’s understanding of interpersonal cognitive processes and the artificial generation of biological motions.

## Data Availability

The raw data supporting the conclusions of this article will be made available by the authors, without undue reservation.

## Author’s note

A part of this paper was presented at the Human Communication Science (HCS) workshop of the Institute of Electronics, Information and Communication Engineers and reported as a preprint in Japanese.

## Supplementary material

Supplementary VIDEO 1 Sample gait animation featuring data from a barefoot nonmodel.

Supplementary VIDEO 2 Sample gait animation featuring data from a runway model wearing high heels.
